# Current status and perspectives of interventional clinical trials for glioblastoma – analysis of ClinicalTrials.gov

**DOI:** 10.1186/s13014-016-0740-5

**Published:** 2017-01-03

**Authors:** Nikola Cihoric, Alexandros Tsikkinis, Giuseppe Minniti, Frank J Lagerwaard, Ulrich Herrlinger, Etienne Mathier, Ivan Soldatovic, Branislav Jeremic, Pirus Ghadjar, Olgun Elicin, Kristina Lössl, Daniel M Aebersold, Claus Belka, Evelyn Herrmann, Maximilian Niyazi

**Affiliations:** 1 Department of Radiation Oncology, Inselspital, Bern University Hospital, University of Bern, 3010 Bern, Switzerland; 2 Department of Genitourinary Oncology, David H. Koch Center for Applied Research of Genitourinary Cancers, The University of Texas MD Anderson Cancer Center, Houston, Texas USA; 3 Unit of Radiation Oncology, Sant’ Andrea Hospital, University Sapienza, and IRCCS Neuromed, Pozzilli (IS), Italy; 4 Department of Radiation Oncology, VU University Medical Center, Amsterdam, The Netherlands; 5 Department of Neurology, Division of Clinical Neurooncology, University of Bonn Medical Center, Bonn, Germany; 6 Faculty of Medicine, University of Belgrade, Belgrade, Serbia; 7 Institute of Lung Diseases, Sremska Kamenica, Serbia and BioIRC Center for Biomedical Research, Kragujevac, Serbia; 8 Department of Radiation Oncology, Charité Universitätsmedizin Berlin, Berlin, Germany; 9 Department of Radiation Oncology, LMU Munich, München, Germany; 10 German Cancer Consortium (DKTK) & German Cancer Research Center (DKFZ), Heidelberg, Germany

**Keywords:** Glioblastoma, Clinicaltrials.gov, Interventional Clinical Trials

## Abstract

The records of 208.777 (100%) clinical trials registered at ClinicalTrials.gov were downloaded on the 19th of February 2016. Phase II and III trials including patients with glioblastoma were selected for further classification and analysis. Based on the disease settings, trials were classified into three groups: newly diagnosed glioblastoma, recurrent disease and trials with no differentiation according to disease setting. Furthermore, we categorized trials according to the experimental interventions, the primary sponsor, the source of financial support and trial design elements. Trends were evaluated using the autoregressive integrated moving average model. Two hundred sixteen (0.1%) trials were selected for further analysis. Academic centers (investigator initiated trials) were recorded as primary sponsors in 56.9% of trials, followed by industry 25.9%. Industry was the leading source of monetary support for the selected trials in 44.4%, followed by 25% of trials with primarily academic financial support. The number of newly initiated trials between 2005 and 2015 shows a positive trend, mainly through an increase in phase II trials, whereas phase III trials show a negative trend. The vast majority of trials evaluate forms of different systemic treatments (91.2%). In total, one hundred different molecular entities or biologicals were identified. Of those, 60% were involving drugs specifically designed for central nervous system malignancies. Trials that specifically address radiotherapy, surgery, imaging and other therapeutic or diagnostic methods appear to be rare. Current research in glioblastoma is mainly driven or sponsored by industry, academic medical oncologists and neuro-oncologists, with the majority of trials evaluating forms of systemic therapies. Few trials reach phase III. Imaging, radiation therapy and surgical procedures are underrepresented in current trials portfolios. Optimization in research portfolio for glioblastoma is needed.

**Electronic supplementary material:**

The online version of this article (doi:10.1186/s13014-016-0740-5) contains supplementary material, which is available to authorized users.

## Introduction

Glioblastoma is the most common and aggressive primary parenchymal brain tumor [1, 2]. The current standard of care is based on maximal safe surgical resection followed by concurrent chemoradiation (CTRT) with temozolomide followed by six months of maintenance chemotherapy, resulting in median survival rates of approximately 15 months [3]. Other than temozolomide, only few agents have shown a clinical benefit to treatment with radiotherapy alone [4].

Furthermore, advanced immunotherapeutic strategies have also emerged, thus far without any significant success [5]. The current evidence-based treatment recommendations for systemic therapy are well summarized in the work from Olson et al. [6].

On review of among others recently reported ASTRO [7] and ESTRO-ACROP guidelines for glioblastomas [8], it would appear that substantial innovations with respect to radiotherapy approaches such as target definition, fractionation and planning as well as delivery techniques are largely lacking, or at least these innovations do not appear to find their way to current guidelines. From this unsubstantiated observation, the question arises whether these components of modern radiotherapy have been an integral part of past or current trials performed in glioblastoma patients.

In search of higher transparency and accessibility to information, several institutions and groups have established publicly available clinical trial registries. Trial registration is being regulated with European and US federal laws as well as international conventions (World Health Organization, WHO) [9, 10]. Registration of all interventional clinical trials is obligatory in the European Union (EU) and the United States (US) and is required by an international consortium of medical journal editors [11]. ClinicalTrials.gov is the largest clinical trial registry with over 200,000 registered trials and a high weekly growth rate of new entries. The registration process and its potential for an in-depth analysis of the clinical trials’ landscape is well described in the literature [12–16]. A detailed description of registered protocol elements can be found at the ClinicalTrials.gov website [17–19]. Due to the nature of ClinicalTrials.gov trial submission process, detailed information on past and present clinical trials can be obtained using the ClinicalTrials.gov registry; usually even more details than reported in the eventual peer-reviewed publication.

The aim of the current study was to investigate in how far radiotherapy innovations, in any aspect, have been an integral part within the setup of past and current clinical trials. In recent years, an abundance of phase I trials have been initiated and completed, however, these trials generally have focused on the addition of systemic components in addition to standard forms of (radiotherapy or surgery) treatment, and not as much on potential innovations in the radiotherapy process. Therefore, we restricted the current analysis to phase II and III clinical trials for glioblastoma, reported in ClinicalTrials.gov in recent years.

## Materials and Methods

### Data acquisition

**Fig. 1:**
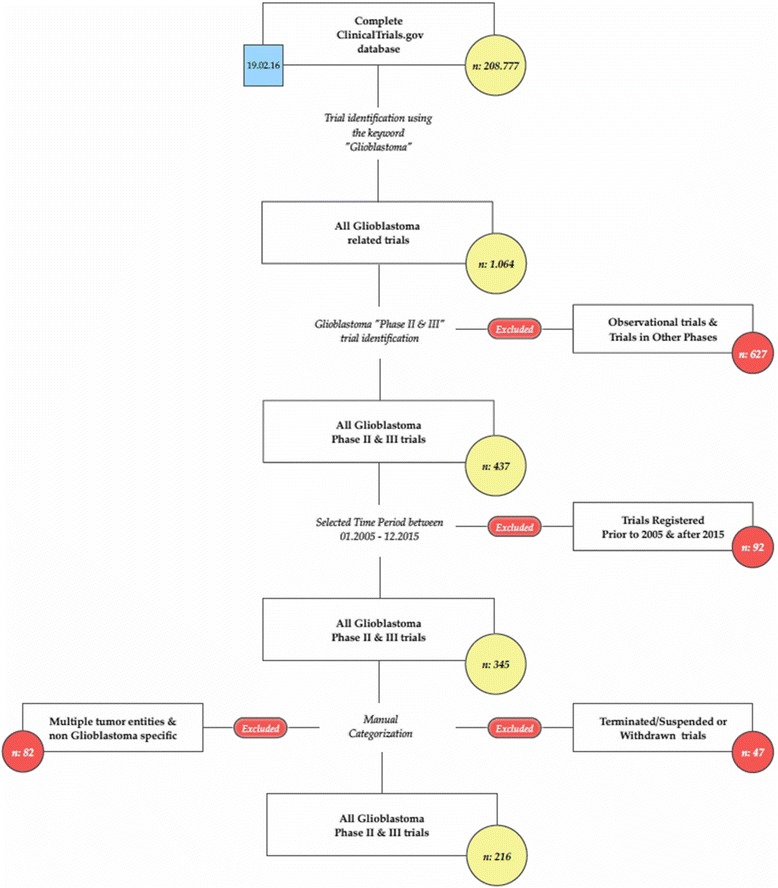
Trials selection process diagram

The records of all 208,777 clinical trials registered at ClinicalTrials.gov were downloaded on the 19th of February 2016 and an SQL database was created to enable further analysis. The following fields were searched for glioblastoma-related keywords (glioblastoma, astrocytoma grade 4 (iv), gliosarcoma, gbm): short title, scientific title, conditions, a short summary and detailed description. Using this search strategy, a total of 1.064 trials were identified. We selected trials registered during the time period from January 2005 to December 2015 for further manual review. After exclusion of prematurely closed, phase I or observational trials and trials not specific for glioblastoma, 216 (20.3%) trials were selected for analysis. The trial selection process is shown in Fig. 1.

Selected trials were divided into three groups: those concerning newly diagnosed glioblastoma, those in recurrent disease and finally trials where it was not specified or both categories were included.

All registered interventions were classified according to their specific role within the trial as part of the standard treatment or the experimental approach. Date of trial registration was taken into account. Systemic treatments were categorized based on the resources available on the following databases: www.drugbank.ca [20], National Cancer Institute Dictionary of Cancer Terms (www.cancer.gov), pubchem.ncbi.nlm.nih.gov as well as the Scopus database, the PubMed Database, Google Scholar and also through a generic internet search (Google search engine). We classified systemic treatments into experimental and approved drugs based on the development status of the specific therapy. Experimental drugs were annotated as such if no previous indication was approved for commercial usage from the United State Food and Drug Administration (FDA). The investigational compounds were further classified based on their specific chemical subgroups according to the World Health Organization ATC/DDD system [21, 22]. By data unavailability, we manually classified the selected drugs according to their chemical group, as suggested in the WHO guideline [22]. Additionally, the specific target receptors and the mechanism of action were also searched and noted.

Radiotherapy, surgical procedures, and imaging procedures were classified according to their role in a clinical trial as part of the standard treatment or as an experimental intervention. If the procedure was in the focus of a particular trial it was considered as an experimental intervention. All surgical, as well as radiotherapy approaches in the recurrent setting, were considered experimental. Finally, all other interventions that do not belong to the mentioned groups, but were evaluated within a trial protocol were also considered as experimental.

In classifying the source of funding we used a modified strategy based on the methodology previously described in the work of Califf et al. [14].

#### Statistical methodology

Forecasting has been performed using the ARIMA (Autoregressive Integrated Moving Average) model. Based on Akaike information criterion (AIC), Root-mean-square deviation (RMSE) and R squared, confidence interval and logical outcome, the best model was chosen. The time interval was 11 years divided into quarters. We used the autoregressive order 1, difference 0 and moving average 1 [23]. Only non-seasonal structure was used. Forecasting was performed using R 3.3.0 statistical software.

## Results

Trial design characteristics are presented in Table 1 and a general overview of the trials is shown in Table 2.

**Table 1:** Trials design data

	Number	Percent
Trial Phase		
Phase 2	188	87.0
Phase 2/Phase 3	3	1.4
Phase 3	25	11.6
Number of Arms		
1	107	49.5
2	83	38.4
≥3	13	9.7
NR	5	2.3
Sample Size		
0 to 50	90	41.7
51 to 100	62	28.7
101 to 200	36	16.7
201 to 300	11	5.1
301 or more	16	7.4
NR	1	0.5
Interventional Model		
Single Group Assignment	116	53.7
Parallel Assignment	92	42.6
Factorial Assignment	1	0.5
Crossover Assignment	2	0.9
NR	5	2.3
Treatment Allocation		
Non-Randomized	47	21.8
Randomized	86	39.8
NR	83	38.4
Masking (Blinding)		
Open Label	187	86.6
Single Blind	2	0.9
Double Blind	25	11.6
NR	2	0.9
Endpoint Classification		
Safety/Efficacy Study	109	50.5
Efficacy Study	75	34.7
Safety Study	4	1.9
Bio-equivalence Study	1	0.5
Pharmacodynamics Study	1	0.5
Pharmacokinetics Study	1	0.5
NR	25	11.6
Primary Purpose of Trial		
Treatment	207	95.8
Diagnostic	3	1.4
Health Services Research	1	0.5
Basic Science	2	0.9
Supportive Care	1	0.5
NR	2	0.9

**Table 2:** Trial characteristics

	Number	Percent
Disease Settings		
1	5	2.3
2	96	44.4
3	115	53.2
Systemic Therapy as Investigative Intervention		
0	19	8.8
1	95	44.0
2	84	38.9
3	17	7.9
4	1	0.5
Radiotherapy as Investigative Intervention		
Not Used	121	56.0
Part of Standard Protocol	75	34.7
Experimental	20	9.3
Imaging as Investigative Intervention		
Not Mentioned^a^	206	95.4
Experimental	10	4.6
Surgery as Investigative Intervention		
Not Used	134	62.0
Part of Standard Protocol	71	32.9
Experimental	11	5.1
Trial Overall Status		
Completed	93	43.1
Active, not recruiting	52	24.1
Not yet recruiting	13	6.0
Recruiting	58	26.9
Primary Sponsor Type		
Industry	56	25.9
NIH	20	9.3
academy	123	56.9
collaborative group	17	7.9
Date of Registration		
2005–2009	101	46.8
2010–2015	115	53.2

^a^Majority of trials do not mention imaging procedures in any contest. Registered Data do not provide possibility to extract information about utilization of imaging procedures as part of standard protocol

**Fig. 2:**
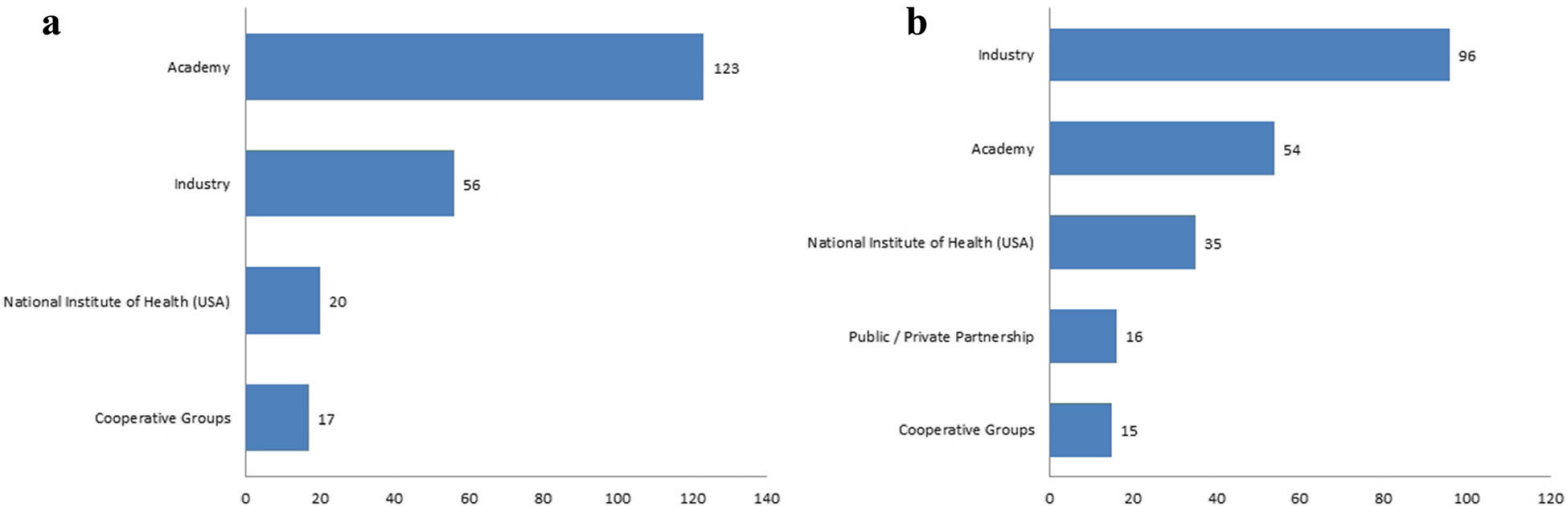
Trials Primary Sponsors (**a**) and Probable Source of Monetary Support (**b**)

Academic centers (investigator-initiated trials) were recorded as primary sponsors in 56.9% of the trials, followed by industry 25.9%. Industry is the leading source of monetary support in 44.4%, followed by academia in 25%. Other sponsors and sources of monetary support are presented in Fig. 2.

**Fig. 3:**
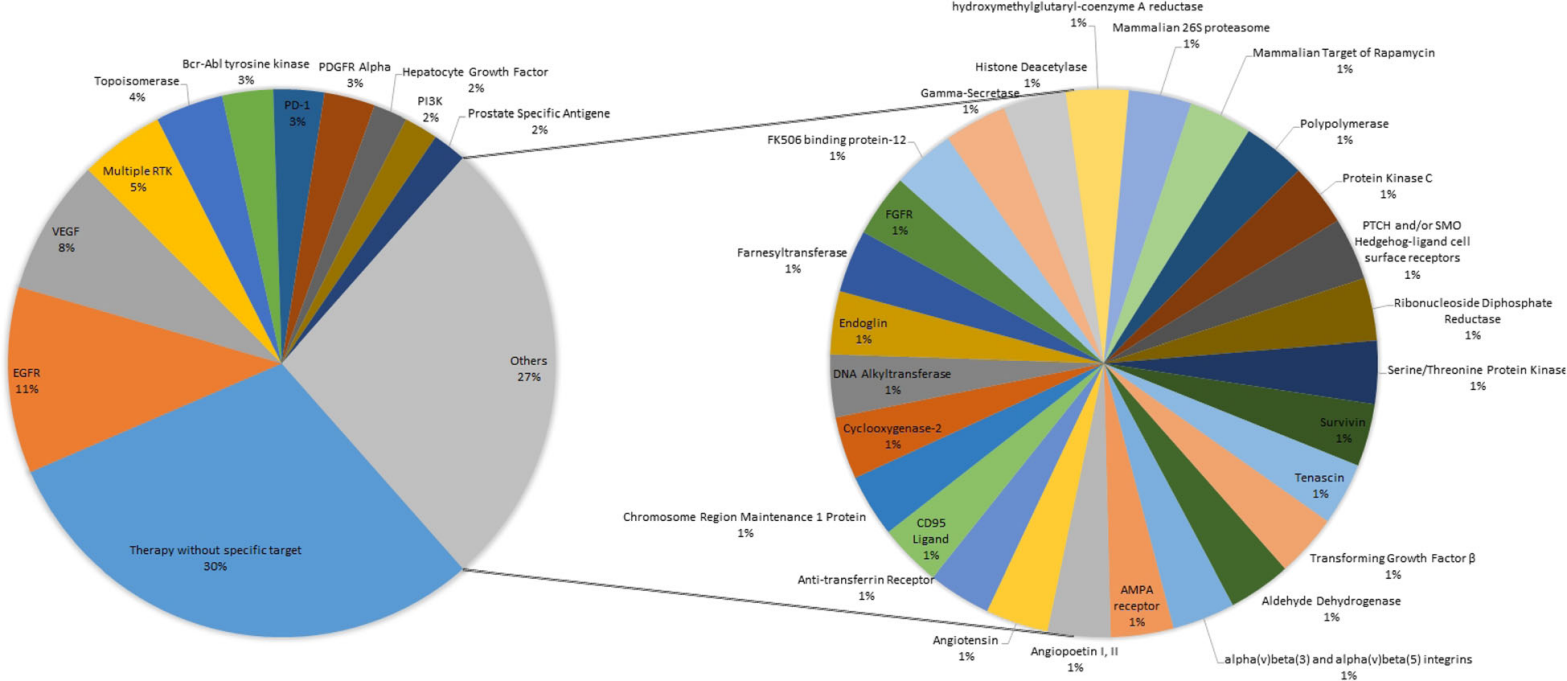
Systemic therapy

The number of yearly initiated trials between 2005 and 2015 ranged between 1 and 11, and shows a slightly positive trend (Fig. 3).

### Systemic therapy

The majority of trials evaluate forms of systemic therapeutic approaches (*n* = 197, 91.2%). 43.7% of those were in primary, 53.8% in recurrent and 2.5% in both disease settings. Majority was phase II (87.8%), followed by phase III (11.2%). 54.3% of trials on systemic therapy was single arm without adequate comparator. In total 100 different molecular agents or biologicals were identified. Of those, 40 (40%) had been initially approved for indications other than glioblastoma or CNS malignancies. Two (2%) were approved for glioblastoma or other CNS or solid malignancies (carmustine, lomustine) and just one (1%), namely temozolomide, for glioblastoma. Of the 57 (57%) compounds with investigational status 37 (37%) had been investigated for other tumors and one for non-malignant conditions (1%). Nineteen (19%) substances were developed specifically for the treatment of glioblastoma.

Classifying by drug type, 67 (67%) substances belong to small molecules, 32 (32%) to biologicals and for one (1%) substance we did not find any data. 42 (42%) compounds were registered in the WHO ACT/DDD database, with the most common group being protein kinase inhibitors (*n* = 14, 14%), followed by “other neoplastic agents” (*n* = 7, 7%), monoclonal antibodies (*n* = 5, 5%) and lastly immunosuppressants and nitrosoureas (each represented with 2 compounds (*n* = 4, 4%)). The last 12 registered products belong to different classes.

The remaining 58 (58%) compounds not registered in the WHO ACT/DDD database most commonly evaluated protein kinase inhibitors (*n* = 11, 11%), followed by monoclonal antibodies (*n* = 13, 13%) of which 5 were conjugated with toxins or radioisotopes and 6 (6%) vaccines. Alkylating agents, DNA repair inhibitors, and other antineoplastic agents were each represented by 2 (4%) substances. Twenty-two (22%) substances belong to other individual classes.

For 30 systemic therapeutic entities, we did not find any specific target receptor or pathway. The most commonly researched targeted therapy aimed the EGFR receptor or its pathway (*n* = 11, 11%), followed by VEGF (*n* = 8, 8%) and multi-TKIs (n = 5, 5%). An overview of all the investigated systemic therapy agents is presented in Additional file 1: Appendix 2. and Fig. 3.

#### Surgery

##### Primary glioblastoma

In the primary setting, surgery belonged to the investigational arm in four trials. Three of these studies reported on the use of 5-aminolevulinic acid (5-ALA) as a guidance help during surgery. One single other study concentrated, among other endpoints, on the cost effectiveness ratio between 5-ALA contrast enhanced surgery versus placebo.

##### Recurrent glioblastoma

Only a single trial (NCT02394626 - RESURGE) concentrated solely on the efficacy of surgery. RESURGE is a randomized phase II trial with aim to evaluate value of surgical resection followed by second line therapy compared with second line therapy alone. The results should serve as a basis for larger phase III trial. In 6 other studies, surgery was part of the combined modality treatment for recurrent GBM in combination with chemotherapeutic strategies.

##### Radiotherapy

Twenty trials (9.3% of all) evaluate RT as experimental interventions. Of those only one (0.5%) was phase III, namely NCT01450449 trial, sponsored by the International Atomic Agency on short (5x5 Gy) vs. standard course (15x2.7 Gy) of RT in elderly patients.

##### Primary glioblastoma

Fifteen (6.9% of all) trials concentrated on radiotherapy in the primary setting, with four (1.9%) trials exploring hypofractionated regimens, six (2.8%) dose escalation, three (1.4%) target volume definition (Subventricular zone RT, delineation with MRI vs. PET, Whole Brain Low Dose RT) with one (0.5%) trial evaluating the Boron-Neutron Capture Therapy and one (0.5%) comparing Intensity-modulated Radiotherapy (IMRT) versus Intensity-modulated Proton Therapy (IMPT).

##### Recurrent glioblastoma

Five (2.3% of all) trials were evaluating the RT in the recurrent setting. Two (0.9%) trials explored bevacizumab with or without radiotherapy where in one trial RT was applied in form of radiosurgery. One (0.5%) combined APG-101 with re-irradiation with a dose of 36 Gy. One (0.5%) evaluates two hypo-fractionated regimen (5x5 Gy vs 5x7 Gy), and one (0.5%) evaluates delineation based on Amino-Acid PET with dose based on MRI.

### Imaging

Six trials evaluate imaging procedures in the primary setting. Of those three evaluated MRI spectroscopy in delineating the radiotherapy volume and two FMISO-PET as a potential predictor. One trial directly compared MRI versus FDG-PET in target volume delineation. Four trials evaluated procedures in recurrent glioblastoma, one comparing MRI versus Amino acid-PET for target delineation, one FET-PET for evaluating the response to bevacizumab, one FMISO-PET, and MRI to evaluate the delivery of bevacizumab and one investigating the value of MRI for target delineation under bevacizumab treatment.

#### Other investigative treatments

Six trials explored the utilization of NovoTTF® device, three Gliadel Wafer®, two transcranial magnetic stimulation, and one the local application of cellular adoptive immunotherapy (Autologous Lymphocytes). One trial explored the feasibility of molecular profiling with whole genome sequencing and one the treatment of anxiety in patients with glioblastoma.

**Fig. 4:**
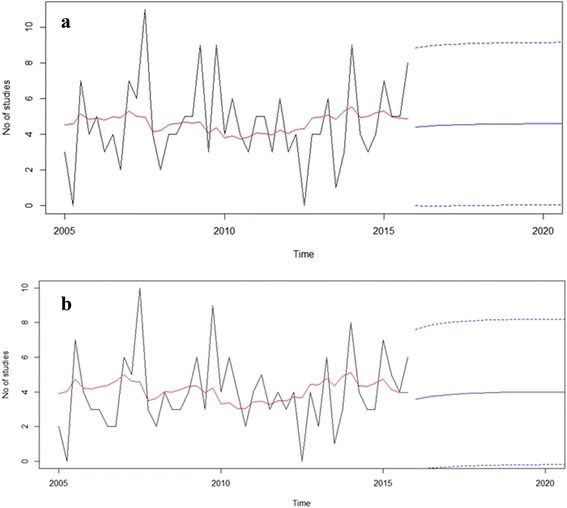
New trials per year. Panel **a** shows ARIMA forecast model for all evaluated trials. Panel **b** shows ARIMA forecast model for phase II trials only

The total number of trials initiated during the period (2005–2015) and forecast (2016–2020) is presented in Fig. 4. In regard to the several examined models, this model showed the optimal AIC (203.52) and RMSE (2.194). As presented in Fig. 2, in the next five years, it is expected that the number of studies show minor increase. Similarly with the previous model, increasing trend is observed regarding to number of phase II studies.

All trials evaluating surgery, radiotherapy, imaging and other investigative treatments are listed in Additional file 2: Appendix 1.

## Discussion

In this work, we analyzed initiatives for trials for primary and recurrent glioblastoma in the last 10 year period. The total number of trials initiated yearly shows a slightly positive trend, although this appears to be mainly driven by a rise in the number of phase II trials, which constitute the vast majority of trials in primary and recurrent glioblastoma, whereby no difference exists in terms of disease setting (reccurent or primary). The most probable cause for this high initiation of trials will probably be the failure of previous early investigative treatments to show a clinical significant advantage. Rather than starting more trials unselectively, patients with (primary and recurrent) glioblastoma should be enrolled in early phase trials with novel designs, such as basket trials where patient selection is based on the genomic profile of the tumor rather than anatomical or classical histological classification [24].

Funding in glioblastoma reflects the situation across all clinical trials in oncology. Bradford et al. found that 41.8% of trials were funded primarily by the industry [25], which corresponds with the 51.9% in our study. A reason for concern should be the lack of industrial funding for research beyond forms of systemic therapy. The industry has displayed a strong interest in sponsoring trials for glioblastoma, and a variety of drugs are already in the investigative pipeline. At the same time, surgery, radiotherapy, and imaging focused trials represent just a small part of the glioblastoma clinical trial portfolio, being investigated in the experimental part in only 9.7% of all phase II/III glioblastoma trials. Research of the aforementioned modalities is supported and driven only from academic institutions while collaborative groups or NIH support is sparse.

It appears that even in the academic community either the interest, or the resources, for initiating of imaging, radiotherapy or surgery research are limited. Of the 123 trials initiated by academic centers, only a minority (19.5% of all GBM academic trials) evaluated these treatment modalities in the experimental setting. Systemic therapies are the focus of investigation in 66% of purely academic trials, without registered or published external financial support. It appears that medical oncologists are leading the way in academic circles with innovative approaches in the treatment of glioblastoma. Based on the available data on ClinicalTrials.gov, we cannot assess whether the required research funding originates from third party sources such as research grants or private donations or from the academic institution.

Trials evaluating exclusively glioblastoma may appear rare when expressed in relative numbers (0.1% of all registered trials). Nevertheless, it is worth mentioning that malignancies of the central nervous system represent a high percentage of the overall cancer trials portfolio and are more often in the research focus compared to other tumor entities with often significantly higher incidences, such as uterine, bladder and esophageal cancer [25].

Surgery in glioblastoma patients is of particular importance and should be evaluated in detail. Trials in the primary setting with surgery in focus, evaluate application of 5-ALA tissue contrast and only one trial evaluates resection optimization but from an economic perspective (NCT01811121). Keeping in mind the poor prognosis of glioblastoma and the predominance of failures at or near the original tumor location, the role of maximal safe surgical resection, lead to better outcome. There is solid data showing that the brain plasticity is more flexible than what was previously thought and therefore a more radical resection could be feasible [26]. The extent of resection is a known prognostic factor that influences survival. Lacroix at al. has shown that the extirpation of 98% or more of gadolinium-enhancing tumor mass was necessary to achieve significant survival [27] and Sanai et al. concluded that at least 78% of the enhancing tumor mass should be resected [28]. Finally, Marko et al. concluded in a recent analysis that any degree of resection is associated with a survival benefit [29]. All three analyses are based on retrospective data and this topic deserves a prospective evaluation. However, it seems that surgery does not belong to the research focus of individual researchers, industry or collaborative groups.

Next well established therapy for glioblastoma is radiation therapy, where the standard dose for primary GMB in younger patients with a good performance status is 60 Gy [3]. For elderly patients hypofractionated radiotherapy results in only a small benefit compared to the best supportive care [30], with the most important prognostic factor being age and the performance status [31].

Trials concentrated on specific radiotherapy questions are mostly in phase II, with a small sample size and initiated mainly through the academy.

As glioblastoma incidence is the highest in older patients, the optimal therapy for this particular group is of high interest. The currently available data suggest that surgical resection may play an important role [32, 33]. Adjuvant therapy is still a subject of investigation. Some data suggest that a combination therapy (RT and temozolomide) may cause excess toxicity [34, 35] and the current evidence suggests that radiotherapy alone should be preferred in patients without MGMT promoter methylation (Methusalem NOA-8 trial). Hypofractionation is an attractive option for this particular patient group [36, 37]. In addition, there is initial evidence that hypofractionated radiotherapy may be combined with temozolomide and result in a better outcome without the excess toxicity [38, 39]. The NCT00482677 trial, could potentially provide definitive answers. Here hypofractionation alone was compared with hypofractionation and temozolomide in a population older than 65 years. The preliminary findings of this trial presented at ASCO 2016 showed a significant benefit for the combined therapy schedule.

Alternative radiotherapy schedules, dose or volume alterations could be of interest for the treatment of glioblastoma regardless of patient age [40], although we currently do not have high-quality prospective data. Moderate dose escalation trials with conventional radiotherapy methods failed to show an improvement in the outcome [41]. Irradiation of the gross tumor volume, even with higher doses, may be insufficient owed perhaps to the infiltrative nature of glioblastoma [42]. Higher dose escalation to the extended volume is not feasible with the standard techniques due to the close proximity of critical structures. The current body of knowledge in regard to the benefits of dose escalation is limited, but an intriguing report on dose escalation comes from Tsien et al. Patients were treated with 66–81 Gy delivered by IMRT with concurrent temozolomide. No grade 3 toxicities were reported and a median survival of 20.1 months was observed [43]. Several trials evaluated dose escalation, with one of them deserving particular mention: “MRSI Guided Dose Escalated Radiation in Glioblastoma” (NCT02394665). Here investigators want to utilize the capabilities of magnetic resonance spectroscopy imaging to detect in addition to the standard MRI and high-risk area at risk of failure, the metabolically active areas in newly diagnosed glioblastoma. Patients will in a first phase undergo standard IMRT with an additional stereotactic radiosurgical boost to the High-Risk Tumor Volume as defined by 3D MRSI [44].

Furthermore, investigators try to evaluate volume alterations together with dose de-escalation in the two Phase II trials namely NCT01822275 and NCT02177578. The tumor location in regard to the Subventricular Zone seems to be of importance [45].

The combination of systemic therapies with radiation as a chemosensitizing agent is certainly attractive. A notable example is trial NCT01071837, where investigators combined re-irradiation (36 Gy/2 Gy ED) with a CD95 ligand inhibitor (APG101). Compared with re-irradiation alone, the combination with APG101 shows a better 6-month progression free survival and slightly better overall survival [46]. But radiosensitization is not a well-understood process and may be dependent on specific cell properties such as genetic alterations. Some tumor cell lines can be resistant to specific radiosensitizers depending on their molecular profile [47]. In addition, glioblastoma evolves during the time, and some passenger mutations may be responsible for resistance development, either towards chemo or radiotherapy.

Several trials are initiated in an effort to answer these questions. The influence of radiotherapy on the clinical course of the disease is certainly of the highest interest. However, there are additional factors, beyond toxicity and efficacy, that should be considered if radiotherapy is applied. Some speculated that radiotherapy may even increase the malignant potential of the tumors [48, 49]. This hypothesis seems to be especially valid for glioblastoma [50, 51]. Accelerated repopulation or selection of more resistant cells is well recognized and described the process in many tumor entities, and it may be that the combination of geographical miss or an underdosing of the volume of interest (recognized or not) plays a role.

Imaging modalities are rarely evaluated in the prospective setting. The most commonly utilized experimental imaging modalities are MRI spectroscopy and FMISO-PET-CT. Besides, an FET-PET and Amino-Acid PET are used for the delineation of radiotherapy treatment volume in recurrent glioblastoma. Imaging is used to predict treatment response or to delineate the volume of interest. All but one trial (NCT01507506) are in phase II with low sample size. Further exploring of this area is necessary. Furthermore, the size and role of the optimal radiotherapy margins are also a matter of debate [52, 53]. This is of particular importance in recurrent glioblastoma, where we have significantly less experience. Evaluation of different imaging modalities in prospective settings may play an important role in reducing this uncertainty [54]. Beside the target delineation, information gathered from different sources, such as MRI, may be used for dose calculations [55]. Furthermore, imaging is very interesting for the evaluation of disease progression and response to the therapy. It may be potentially used for the patient selection that will respond well to the systemic therapy [56, 57].

Although some research activity in imaging and radiotherapy is present, this is not enough, especially if compared with trials that evaluate systemic approaches. Current margins of 1.5-2 cm and a RT dose concept for GBM (60 Gy in 2 Gy fraction) are based on relapse pattern analyses and toxicity profiles from a pre-systemic era. If those are still necessary remains unclear and it will be hardly possible to change current practice on a larger scale without phase III data. However we did not detect any trials to address these specific questions.

The majority of evaluated systemic therapies target specific receptors or pathways. Beside protein kinase inhibitors, which represent the majority of evaluated drugs, we detected over 20 different substances that try to target other pathways and also 30 substances that do not aim a specific pathway, mostly cytotoxic agents. Interesting findings of this study is that 57% of the investigated substances were not approved for marketing in any tumor entities, but only 24% were initially intended for use in glioblastoma or CNS malignancies. As the majority of those are sponsored or supported by the industry, this indicates that glioblastoma is an attractive target even financially. A small benefit in any of the endpoints may provide significant marketing advantages and possibly profit, even when accounting the low overall incidence of glioblastoma. Currently, there is only two systemic therapies approved for glioblastoma two from the cytotoxic group and one from the targeted group. Glioblastoma is also an attractive target from a clinical trial workflow perspective. Short term follow-up is certainly reducing the overall cost of the trial [58].

It seems that immunotherapy is a promising treatment modality and the combination with other options could potentially have a synergistic effect. The potential of other therapy forms to induce an immune response is also recognized. One important preclinical study has already shown that fractionated radiation may induce cell death [59]. This should be evaluated further in order to better understand the complex processes between the immune system and CNS and tumor interactions.

Nevertheless results of phase II trials should always be regarded cautiously. The problem of promising therapies with consequent failures in pivotal studies is recognized. An improvement of the trial design would be beneficial to all involved parties [60].

At the time of analysis, the majority of trials were completed with 43.1% (*n* = 93) trials reported as completed and 24.1% (*n* = 52) with achieved recruitment goals. Even though the results for 39 (18.1%) trials were reported, only 16 of them were accompanied by their publication on ClinicalTrials.gov. This could be attributed to several possible causes: Some of the published work was not captured by an automatic search of the PubMed database [61]. The second reason is the time required from finishing a trial to final publication, in some cases exceeding 30 months [62]. Some trials will also never reach publication for unknown reasons [63], with a significant proportion possibly attributed to negative result and as a result a lack of interest either by the editors or the authors.

### Limitations

The presented analysis is not without its limitations. Some data might have been incorrectly registered in the ClinicalTrials.gov. Moreover, we cannot exclude the possibility that certain data were misclassified during the selection and classification. In addition, the data sets for all trials in the database are not always complete and up-to-date. However, we took great care to minimize these limitations: two authors (NC and AT) crosschecked all trials identified and the trial selection steps.

Additionally, there may be a bias in in terms of number of registered trials in recent years compared with early period. We believe that our analysis is unique and important and that it provides researchers and clinicians with a realistic picture of the future of glioblastoma treatment.

## Conclusions

Investigation in glioblastoma is mainly driven or sponsored by the industry and medical oncologists, with the majority of trials evaluating forms of systemic therapies. Most of the trials are in phase II with just a few trials ever reaching phase III. Imaging, surgery and radiation therapy are heavily underrepresented treatment methods in terms of investigations. Optimization in research portfolio for glioblastoma is needed.

## Additional files

Additional file 1: Appendix 2. Systemic agents investigated for treatment of glioblastoma. (DOCX 19 kb)
Additional file 2: Appendix 1. Trials that evaluate surgery, radiotherapy, imaging and other treatment modalities. (DOCX 23 kb)

## Abbreviations

5-ALA: 5-aminolevulinic acid
ADC: Apparent diffusion coefficient
AGT: O6-alkylguanine-DNA alkyltransferase
AIC: Akaike information criterion
ATC Classification system: Anatomical Therapeutic Chemical classification system
BBB: Blood–brain Barrier
BNCT: Boron neutron capture therapy
CI: Confidence Interval
CNS: Central Nervous System
CR: Complete Response
CSF: Cerebral Spinal Fluid
CTCAE: Common Terminology Criteria for Adverse Events
DCE: Dynamic Contrast-Enhanced
DCs: Dendritic cells
DD: Dose-dense
DDD: Defined Daily Dose
DI: Dose Intensified
DNA: Deoxyribonucleic acid
ECOG: Eastern Cooperative Oncology Group
EGFR: Epidermal growth factor receptor
EIAEDs: CYP3-A inducing anti-epileptics
EORTC: European Organisation for Research and Treatment of Cancer
EU: European Union
FDA: Food and Drug Administration
FDG: Fluorodeoxyglucose
FET: Fluoro-ethyl-tyrosin
Flt-3: Fms-like tyrosine kinase 3
FMISO: Fluoro misonidazole
GBM: Glioblastoma
G-CIMP: Glioma-CpG island methylator phenotype
Gy: Gray
HR: Hazard ratio
IMPT: Intensity-modulated Proton Therapy
IMRT: Intensity-mModulated Radiotherapy
KLH: Keyhole limpet hemocyanin
KPS: Karnofsky Performance Status
LAK: Lymphokine-activated killer
MGMT: Methyl Guanine Methyl Transferase
MMP: matrix metalloproteinase
MRI: Magnetic Resonance Imaging
mRNA: Messenger ribonucleic acid
NCF: Neurocognitive function
NCI: National Cancer Institute
NCI: National Cancer Institute
O6-BG: O6-benzylguanine
OS: Overall Survival
pAKT: Phosphorylated Protein Kinase Strain
PDGFR: Platelet-derived growth factor receptors
PET: Positron emission tomography
PFS: Progression-free survival
Poly-ICLC: Poly-l-lysine and carboxymethylcellulose
PR: Partial Response
PTEN: Phosphatase and tensin homolog
QOL: Quality of life
RMSE: Root-mean-square deviation
RPA: Recursive partitioning analysis
RT: Radiotherapy
RTOG: Radiation Therapy Oncology Group
STD: Standard Dose
TKI: Tyrosine Kinase Inhibitor
TTP: Time to progression
US: United States
VEGF: Vascular endothelial growth factor
VEGFR: Vascular endothelial growth factor receptor
WHO: World Health Organization
