# Supplementary material for: Current status and perspectives of interventional clinical trials for glioblastoma – analysis of ClinicalTrials.gov
Source: Radiat Oncol. 2017 Jan 3;12:1. doi: 10.1186/s13014-016-0740-5 (PMC5210306; doi:10.1186/s13014-016-0740-5)
Supplement: Additional file 1: Appendix 2. — Systemic agents investigated for treatment of glioblastoma. (DOCX 19 kb) [file 13014_2016_740_MOESM1_ESM.docx]

| compound | Status | Recognized Indication / Also Investigated For | Drug Class / Chemical Entity | Targeted Receptor or Pathway |
| --- | --- | --- | --- | --- |
| ABT-414 | INV | Glioblastoma | Monoclonal Antibody, Conjugated | EGFR |
| Aldoxorubicin | INV | Solid Or Hematological Malignancies | Anthracyclines | NO |
| APG101 | INV | Solid Or Hematological Malignancies | Human Fusion Protein | CD95 Ligand |
| AR-67 | INV | Glioblastoma | Topoisomerase Inhibitors | Topoisomerase |
| Ascorbic Acid | APP | Non-Malignant Conditions | Nutraceutical | NO |
| Atorvastatin | APP | Non-Malignant Conditions | HMG CoA reductase inhibitors | hydroxymethylglutaryl-coenzyme A reductase |
| Axitinib | APP | Solid Or Hematological Malignancies | Protein Kinase Inhibitor | VEGF |
| Bevacizumab | APP | Solid Or Hematological Malignancies | Monoclonal Antibody | VEGF |
| BGJ398 | INV | Glioblastoma | Protein Kinase Inhibitor | FGFR |
| Bortezomib | APP | Solid Or Hematological Malignancies | Other Antineoplastic Agents | Mammalian 26S proteasome |
| Bosutinib | APP | Solid Or Hematological Malignancies | Protein Kinase Inhibitor | Bcr-Abl tyrosine kinase |
| Buparlisib (BKM120) | INV | Solid Or Hematological Malignancies | Lipid Kinase Inhibitor | PI3K |
| Cabazitaxel | APP | Solid Or Hematological Malignancies | Taxanes | NO |
| Cabozantinib | APP | Solid Or Hematological Malignancies | Protein Kinase Inhibitor | VEGF |
| Carmustine | APP | Glio/Cns/Other | Nitrosoureas | NO |
| Cediranib | INV | Solid Or Hematological Malignancies | Protein Kinase Inhibitor | VEGF |
| Celecoxib | APP | Non-Malignant Conditions | Other Antineoplastic Agents | NO |
| Cetuximab | APP | Solid Or Hematological Malignancies | Monoclonal Antibody | EGFR |
| Chloroquine | APP | Non-Malignant Conditions | Aminoquinolines | NO |
| Cilengitide | INV | Glio/Cns/Other | Integrin Inhibitors | alpha(v)beta(3) and alpha(v)beta(5) integrins |
| Cotara | INV | Glioblastoma | Monoclonal Antibody, Conjugated | NO |
| CpG-ODN | INV | Glioblastoma | NO DATA AVAILABLE | NO |
| crenolanib | INV | Solid Or Hematological Malignancies | Protein Kinase Inhibitor | PDGFR Alpha |
| CT-322 (pegdinetanib) | INV | Glioblastoma | Peptide | VEGF |
| Cyclophosphamide | APP | Solid Or Hematological Malignancies | Nitrogen Mustard Analogues | NO |
| dacomitinib | INV | Glioblastoma | Protein Kinase Inhibitor | EGFR |
| dasatinib | APP | Solid Or Hematological Malignancies | Protein Kinase Inhibitor | Multiple RTK |
| dendritic cells vaccine | INV | Glioblastoma | Vaccine | NO |
| disulfiram | APP | Non-Malignant Conditions | Drugs used in alcohol dependence | Aldehyde Dehydrogenase |
| Dovitinib | INV | Solid Or Hematological Malignancies | Protein Kinase Inhibitor | Multiple RTK |
| Enzastaurin | INV | Solid Or Hematological Malignancies | Protein Kinase Inhibitor | Protein Kinase C |
| Epothilone | INV | Solid Or Hematological Malignancies | polyketide | NO |
| ERC1671 vaccine | INV | Glioblastoma | Vaccine | NO |
| Erlotinib | APP | Solid Or Hematological Malignancies | Protein Kinase Inhibitor | EGFR |
| Etoposide | APP | Solid Or Hematological Malignancies | Podophyllotoxin derivatives | Topoisomerase |
| Everolimus | APP | Solid Or Hematological Malignancies | Protein Kinase Inhibitor | FK506 binding protein-12 |
| Fotemustine | APP | Solid Or Hematological Malignancies | Alkylating Agent | NO |
| GDC-0449 (Vismodegib) | APP | Solid Or Hematological Malignancies | Other Antineoplastic Agents | PTCH and/or SMO Hedgehog-ligand cell surface receptors |
| Gefitinib | APP | Solid Or Hematological Malignancies | Protein Kinase Inhibitor | EGFR |
| gossypol | INV | Solid Or Hematological Malignancies | Phenoles | NO |
| HSPPC-96 Vaccine | INV | Glio/Cns/Other | Vaccine | NO |
| Hydroxyurea | APP | Solid Or Hematological Malignancies | Other Antineoplastic Agents | ribonucleoside diphosphate reductase |
| Imatinib | APP | Solid Or Hematological Malignancies | Protein Kinase Inhibitor | Bcr-Abl tyrosine kinase |
| INNOCELL Immuncell-LC | INV | Glioblastoma | Activated T lymphocyte | NO |
| Irinotecan | APP | Solid Or Hematological Malignancies | Other Antineoplastic Agents | Topoisomerase |
| Isotretinoin | APP | Non-Malignant Conditions | Retinoids for treatment of acne | NO |
| lapatinib | APP | Solid Or Hematological Malignancies | Protein Kinase Inhibitor | EGFR |
| lenalidomide | APP | Solid Or Hematological Malignancies | Other immunosuppressants | Cyclooxygenase-2 |
| lomustine | APP | Glio/Cns/Other | Nitrosoureas | NO |
| losartan | APP | Non-Malignant Conditions | Angiotensin II antagonists plain | Angiotensin |
| LY2157299 | INV | Glio/Cns/Other | Protein Kinase Inhibitor | Transforming Growth Factor β |
| MEDI4736 | INV | Solid Or Hematological Malignancies | Monoclonal Antibody | PD-1 |
| MEDI-575 | INV | Solid Or Hematological Malignancies | Monoclonal Antibody | PDGFR Alpha |
| Methoxyamine | INV | Solid Or Hematological Malignancies | DNA Repair Inhibitor | DANN |
| Mipsagargin | INV | Solid Or Hematological Malignancies | Mitotic Inhibitor | PSA |
| MPC-6827 | INV | Glioblastoma | Other Antineoplastic Agents | NO |
| Neuradiab | INV | Glioblastoma | Monoclonal Antibody, Conjugated | Tenascin |
| Nimotuzumab | INV | Solid Or Hematological Malignancies | Monoclonal Antibody | EGFR |
| Nivolumab | APP | Solid Or Hematological Malignancies | Monoclonal Antibody | PD-1 |
| O6-benzylguanine | INV | Glio/Cns/Other | Guanin Analog | DNA Alkyltransferase |
| Olaratumab | INV | Glioblastoma | Monoclonal Antibody | PDGFR Alpha |
| Onartuzumab | INV | Solid Or Hematological Malignancies | Monoclonal Antibody | Hepatocyte Growth Factor |
| ONC201 | INV | Solid Or Hematological Malignancies | Protein Kinase Inhibitor | Serine/Threonine Protein Kinase |
| paclitaxel poliglumex | INV | Solid Or Hematological Malignancies | Taxanes | NO |
| Panzem | INV | Solid Or Hematological Malignancies | Estradiol Metabolite | NO |
| Pazopanib | APP | Solid Or Hematological Malignancies | Protein Kinase Inhibitor | VEGF |
| pembrolizumab | APP | Solid Or Hematological Malignancies | Monoclonal Antibody | PD-1 |
| PEP-3 vaccine | INV | Glioblastoma | Vaccine | EGFR |
| PLX3397 | INV | Solid Or Hematological Malignancies | Protein Kinase Inhibitor | Multiple RTK |
| Poly ICLC | INV | Solid Or Hematological Malignancies | Other Immunostimulans | NO |
| ponatinib | APP | Solid Or Hematological Malignancies | Protein Kinase Inhibitor | Bcr-Abl tyrosine kinase |
| procarbazine | APP | Solid Or Hematological Malignancies | Methylhydrazines | NO |
| PSMA ADC | INV | Solid Or Hematological Malignancies | Monoclonal Antibody, Conjugated | PSA |
| PX-866 | INV | Glio/Cns/Other | Protein Kinase Inhibitor | PI3K |
| Ramucirumab | APP | Solid Or Hematological Malignancies | Monoclonal Antibody | VEGF |
| Rilotumumab | INV | Solid Or Hematological Malignancies | Monoclonal Antibody | Hepatocyte Growth Factor |
| Rindopepimut | INV | Glioblastoma | Vaccine | EGFR |
| RO4929097 | INV | Solid Or Hematological Malignancies | Gamma-Secretase inhibitor | Gamma-Secretase |
| Selinexor | INV | Solid Or Hematological Malignancies | DNA Repair Inhibitor | Chromosome Region Maintenance 1 Protein |
| SGT-53 | INV | Solid Or Hematological Malignancies | Monoclonal Antibody, Conjugated | Anti-transferrin Receptor |
| Sirolimus | APP | Non-Malignant Conditions | Selective immunosuppressant’s | NO |
| Sorafenib | APP | Solid Or Hematological Malignancies | Protein Kinase Inhibitor | Multiple RTK |
| Sunitinib | APP | Solid Or Hematological Malignancies | Protein Kinase Inhibitor | Multiple RTK |
| SVN53-67/M57-KLH peptide vaccine | INV | Glioblastoma | Vaccine | Survivin |
| Sym004 | INV | Solid Or Hematological Malignancies | Monoclonal Antibody | EGFR |
| Talampanel | INV | Non-Malignant Conditions | AMPA receptor antagonist | AMPA receptor |
| Temozolomide | APP | Glioblastoma | Alkylating Agent | NO |
| Temsirolimus | APP | Solid Or Hematological Malignancies | Protein Kinase Inhibitor | mammalian target of rapamycin |
| TH-302 | INV | Solid Or Hematological Malignancies | Alkylating Agent | NO |
| Thalidomide | APP | Non-Malignant Conditions | Other immunosuppressants | NO |
| Tipifarnib | INV | Solid Or Hematological Malignancies | Farnesyltransferase inhibitor | Farnesyltransferase |
| Tivozanib | INV | Solid Or Hematological Malignancies | Protein Kinase Inhibitor | VEGF |
| Topotecan | APP | Solid Or Hematological Malignancies | Other Antineoplastic Agents | Topoisomerase |
| TP-38 | INV | Glioblastoma | Protein - Toxine Conjugate | EGFR |
| TRC105 | INV | Solid Or Hematological Malignancies | Monoclonal Antibody | Endoglin |
| Trebananib | INV | Solid Or Hematological Malignancies | Peptibody | Angiopoetin I, II |
| VB-111 | INV | Glioblastoma | Gene Based Biological | NO |
| Veliparib | INV | Solid Or Hematological Malignancies | Polymerase Inhibitor | Polypolymerase |
| Verubulin | INV | Glioblastoma | Other Antineoplastic Agents | NO |
| Vorinostat | APP | Solid Or Hematological Malignancies | Other Antineoplastic Agents | Histone Deacetylase |

APP – Approved Drug. Drug may be approved for indications other than GBM.

INV – Investigational Drug. Drug did not gained marketing approval from FDA.
