# Supplementary material for: Current status and perspectives of interventional clinical trials for glioblastoma – analysis of ClinicalTrials.gov
Source: Radiat Oncol. 2017 Jan 3;12:1. doi: 10.1186/s13014-016-0740-5 (PMC5210306; doi:10.1186/s13014-016-0740-5)
Supplement: Additional file 2: Appendix 1. — Trials that evaluate surgery, radiotherapy, imaging and other treatment modalities. (DOCX 23 kb) [file 13014_2016_740_MOESM2_ESM.docx]

| NCT Trial ID | Trial Start Date | Trial brief title | Type of experimental approach – Surgery |
| --- | --- | --- | --- |
| NCT02394626 | 15-05-15 | *Surgery for Recurrent Glioblastoma* | Recurrent GBM Surgery |
| NCT01814813 | 15-05-13 | *Vaccine Therapy With Bevacizumab Versus Bevacizumab Alone in Treating Patients With Recurrent Glioblastoma Multiforme That Can Be Removed by Surgery* | Recurrent GBM Surgery |
| NCT01975701 | 15-12-13 | *A Phase 2 Study of BGJ398 in Patients With Recurrent GBM* | Recurrent GBM Surgery |
| NCT00112866 | 15-01-05 | *Cilengitide in Treating Patients Who Are Undergoing Surgery for Recurrent or Progressive Glioblastoma Multiforme* | Recurrent GBM Surgery |
| NCT00980343 | 15-02-10 | *GDC-0449 in Treating Patients With Recurrent Glioblastoma Multiforme That Can Be Removed by Surgery* | Recurrent GBM Surgery |
| NCT01122901 | 15-12-10 | *Gamma-Secretase/Notch Signaling Pathway Inhibitor RO4929097 in Treating Patients With Recurrent or Progressive Glioblastoma* | Recurrent GBM Surgery |
| NCT00104091 | 15-12-04 | *Safety and Efficacy Study to Treat Recurrent Grade 4 Malignant Brain Tumors* | Recurrent GBM Surgery |
| NCT01006044 | 15-10-09 | *Efficacy & Safety of Autologous Dendritic Cell Vaccination in Glioblastoma Multiforme After Complete Surgical Resection* | Surgery Guided With Tissue Contrast |
| NCT00752323 | 15-08-08 | *Imaging Procedure Using ALA in Finding Residual Tumor in Grade IV Malignant Astrocytoma* | Surgery Guided With Tissue Contrast |
| NCT01310868 | 15-05-11 | *Gliadel Wafer and Fluorescence-Guided Surgery With 5-ALA Followed by Radiation Therapy And Temozolomide in Treating Patients With Primary Glioblastoma* | Surgery Guided With Tissue Contrast |
| NCT01811121 | 15-02-13 | *Medico-Economic Evaluation Of Surgery Guided By Fluorescence For The Optimization Of Resection Of Glioblastomas* | Surgery Guided With Tissue Contrast - Economical Evaluation |

# Trials where surgery is considered as experimental

# Trials where radiotherapy is considered as experimental

| NCT Trial ID | Trial Start Date | Trial brief title | Type of experimental approach – Radiotherapy |
| --- | --- | --- | --- |
| NCT01450449 | 15-02-09 | *Short Course vs. Standard Course Radiotherapy in Elderly and/or Frail Patients With Glioblastoma Multiforme* | Altered Fractionation - Hypofractionation |
| NCT01508117 | 15-01-12 | *Phase II Axitinib (AG-013736) in Elderly Glioblastoma Multiforme (GBM) Patients* | Altered Fractionation - Hypofractionation |
| NCT02206230 | 15-08-14 | *Trial of Hypofractionated Radiation Therapy for Glioblastoma* | Altered Fractionation - Hypofractionation |
| NCT01209442 | 15-08-10 | *Hypofractionated Intensity-Modulated Radiation Therapy With Temozolomide and Bevacizumab for Glioblastoma Multiforme* | Altered Fractionation - Hypofractionation |
| NCT00974987 | 15-09-09 | *Boron Neutron Capture Therapy, Radiation Therapy, and Temozolomide in Treating Patients With Newly Diagnosed Glioblastoma Multiforme* | Boron-Neutron Capture Therapy |
| NCT01507506 | 15-03-11 | *Phase III Study Comparing 2 Brain Conformational Radiotherapy in Combination With Chemotherapy in the Treatment of Glioblastoma* | Dose Escalation to 72 Gy with Simultaneous Integrated Boost |
| NCT00820963 | 15-07-06 | *Standard Radiation Therapy, Higher-Dose Radiation Therapy, or Chemotherapy in Treating Older Patients With Glioblastoma Multiforme* | Dose Escalation |
| NCT02394665 | 15-03-15 | *MRSI Guided Dose Escalated Radiation in Glioblastoma* | Dose Escalation |
| NCT01165671 | 15-07-10 | *Carbon Ion Radiotherapy for Primary Glioblastoma* | Dose Escalation Carbon Ion Boost 6x3 Gy vs Proton RT conventional dose |
| NCT02179086 | 15-11-14 | *Dose-Escalated Photon IMRT or Proton Beam Radiation Therapy Versus Standard-Dose Radiation Therapy and Temozolomide in Treating Patients With Newly Diagnosed Glioblastoma* | Dose Escalation IMRT or Proton RT |
| NCT00253448 | 15-12-02 | *Stereotactic Radiosurgery and Radiation Therapy in Treating Patients With Glioblastoma Multiforme* | Dose Escalation Radiosurgery |
| NCT01854554 | 15-05-13 | *Glioblastoma Multiforme (GBM) Proton vs. IMRT* | IMPT vs IMRT |
| NCT01730950 | 15-12-12 | *Bevacizumab With or Without Radiation Therapy in Treating Patients With Recurrent Glioblastoma* | Re-irradiation 2 weeks dose unknown |
| NCT01071837 | 15-12-09 | APG101 in Glioblastoma | Re-irradiation 36 Gy /2 Gy |
| NCT01464177 | 15-10-11 | Hypofractionated Stereotactic Radiotherapy in Recurrent Glioblastoma Multiforme | Re-irradiation Altered Fractionation Hypo |
| NCT02120287 | 15-05-14 | Border Zone Stereotactic Radiosurgery With Bevacizumab in Patients With Glioblastoma Multiforme | Re-irradiation Stereotactic Radiosurgery |
| NCT01252459 | 15-07-11 | Amino-acid PET Versus MRI Guided Re-irradiation in Patients With Recurrent Glioblastoma Multiforme | Re-irradiation Volume Definition |
| NCT02177578 | 15-05-14 | Subventricular Zone (SVZ) and Temozolomide in Glioblastoma Multiforme | Volume Definition - SVZ |
| NCT01083719 | 15-04-10 | A Comparison of FDG-PET Versus MRI Based Target Volume Delineation in Glioblastoma and the Role of FDG-PET/CT in the Alteration of MRI Based Target Volumes. | Volume Definition – FDG/PET CT vs MRI |
| NCT01822275 | 15-05-13 | Phase II Trial of Low-Dose Whole Brain Radiotherapy With Concurrent Temozolomide and Adjuvant Temozolomide in Patients With Newly-Diagnosed Glioblastoma Multiforme | Volume Definition - Whole Brain RT |

# Trials where radiotherapy is considered as experimental

| NCT Trial ID | Trial Start Date | Trial brief title | Type of experimental approach – Imaging |
| --- | --- | --- | --- |
| NCT00253448 | 15-08-11 | *Stereotactic Radiosurgery and Radiation Therapy in Treating Patients With Glioblastoma Multiforme* | Radiotherapy Volume Delineation |
| NCT00902577 | 15-12-15 | *MRI and PET Scan Using 18F-Fluoromisonidazole In Assessing Tumor Hypoxia in Patients With Newly Diagnosed Glioblastoma Multiforme* | Prediction Of Response |
| NCT00906893 | 15-02-13 | *Evaluation of [18F]-FMISO for Non Operated Glioblastoma* | Prediction Of Response, Therapy Modification |
| NCT01083719 | 15-03-10 | *A Comparison of FDG-PET Versus MRI Based Target Volume Delineation in Glioblastoma and the Role of FDG-PET/CT in the Alteration of MRI Based Target Volumes.* | Radiotherapy Volume Delineation |
| NCT01252459 | 15-12-10 | *Amino-acid PET Versus MRI Guided Re-irradiation in Patients With Recurrent Glioblastoma Multiforme* | Radiotherapy Volume Delineation |
| NCT01756352 | 15-01-16 | *FET-PET for Evaluation of Response of Recurrent GBM to Avastin* | Prediction Of Response To Bevacizumab |
| NCT02076152 | 15-05-15 | *FMISO PET Study of Glioblastoma* | Delivery Of Bevacizumab |
| NCT02120287 | 15-01-16 | *Border Zone Stereotactic Radiosurgery With Bevacizumab in Patients With Glioblastoma Multiforme* | Radiotherapy Volume Delineation, Bevacizumab |
| NCT01507506 | 15-09-14 | *Phase III Study Comparing 2 Brain Conformational Radiotherapy in Combination With Chemotherapy in the Treatment of Glioblastoma* | Radiotherapy Volume Delineation |
| NCT02394665 | 15-08-15 | *MRSI Guided Dose Escalated Radiation in Glioblastoma* | Radiotherapy Volume Delineation |

# Trials where other experimental approach were evaluated

| NCT Trial ID | Trial Start Date | Trial Brief Title | Type Of Experimental Approach |
| --- | --- | --- | --- |
| NCT02623231 | 15-12-15 | *The Effect of Escitalopram on Mood, Quality of Life and Cognitive Functioning in Glioblastoma Patients* | Treatment Of Anxiety |
| NCT00362921 | 15-04-04 | *Gliadel Wafer and O6-Benzylguanine in Treating Patients With Recurrent Glioblastoma Multiforme* | Gliadel Wafer |
| NCT01186406 | 15-04-11 | *Gliadel, XRT, Temodar, Avastin Followed by Avastin, Temodar for Newly Diagnosed Glioblastoma Multiforme (GBM)* | Gliadel Wafer |
| NCT01310868 | 15-05-11 | *Gliadel Wafer and Fluorescence-Guided Surgery With 5-ALA Followed by Radiation Therapy And Temozolomide in Treating Patients With Primary Glioblastoma* | Gliadel Wafer |
| NCT02302235 | 15-02-14 | *Ketogenic Diet Treatment Adjunctive to Radiation and Chemotherapy in Glioblastoma Multiforme: a Pilot Study* | Ketogenic Diet |
| NCT02060890 | 15-09-14 | *A Pilot Trial Testing the Feasibility of Molecular Profiling in Recurrent/Progressive Glioblastoma* | Molecular Profiling |
| NCT01954576 | 15-10-13 | *NovoTTF Therapy in Treating Patients With Recurrent Glioblastoma Multiforme* | NovoTTF |
| NCT01894061 | 15-06-13 | *NovoTTF-100A With Bevacizumab (Avastin) in Patients With Recurrent Glioblastoma* | NovoTTF |
| NCT02348255 | 15-01-16 | *NovoTTF-100A With Bevacizumab and Carmustine in Treating Patients With Glioblastoma Multiforme in First Relapse* | NovoTTF |
| NCT02343549 | 15-01-15 | *A Phase II Study of Optune (NovoTTF) in Combination With Bevacizumab (BEV) and Temozolomide (TMZ) in Patients With Newly Diagnosed Unresectable Glioblastoma (GBM)* | NovoTTF |
| NCT00916409 | 15-06-09 | *Effect of NovoTTF-100A Together With Temozolomide in Newly Diagnosed Glioblastoma Multiforme (GBM)* | NovoTTF |
| NCT00379470 | 15-09-06 | *Effect of NovoTTF-100A in Recurrent Glioblastoma Multiforme (GBM)* | NovoTTF |
| NCT00331526 | 15-02-99 | *Cellular Adoptive Immunotherapy in Treating Patients With Glioblastoma Multiforme* | Therapeutic Autologous Lymphocytes |
| NCT02474966 | 15-11-14 | *Effect of Deep TMS on the Permeability of the BBB in Patients With Glioblastoma Multiforme: a Pilot Study* | Transcranial Magnetic Stimulation |
| NCT02283944 | 15-01-15 | *TMS Electrochemotherapy for Glioblastoma Multiforme* | Transcranial Magnetic Stimulation |
